# Spiro-fused carbohydrate oxazoline ligands: Synthesis and application as enantio-discrimination agents in asymmetric allylic alkylation

**DOI:** 10.3762/bjoc.12.18

**Published:** 2016-01-29

**Authors:** Jochen Kraft, Martin Golkowski, Thomas Ziegler

**Affiliations:** 1 Institute of Organic Chemistry, University of Tuebingen, Auf der Morgenstelle 18, 72076 Tuebingen, Germany; 2 Department of Pharmacology, University of Washington, 1959 NE Pacific St, Box 357280, Seattle, WA 98195, USA

**Keywords:** asymmetric catalysis, carbohydrates, oxazolines, palladium, spiro compounds

## Abstract

In the present work, we describe a convenient synthesis of spiro-fused D-*fructo*- and D-*psico*-configurated oxazoline ligands and their application in asymmetric catalysis. The ligands were synthesized from readily available 3,4,5-tri-*O*-benzyl-1,2-*O*-isopropylidene-β-D-fructopyranose and 3,4,5-tri-*O*-benzyl-1,2-*O*-isopropylidene-β-D-psicopyranose, respectively. The latter compounds were partially deprotected under acidic conditions followed by condensation with thiocyanic acid to give an anomeric mixture of the corresponding 1,3-oxazolidine-2-thiones. The anomeric 1,3-oxazolidine-2-thiones were separated after successive benzylation, fully characterized and subjected to palladium catalyzed Suzuki–Miyaura coupling with 2-pyridineboronic acid *N*-phenyldiethanolamine ester to give the corresponding 2-pyridyl spiro-oxazoline (PyOx) ligands. The spiro-oxazoline ligands showed high asymmetric induction (up to 93% ee) when applied as chiral ligands in palladium-catalyzed allylic alkylation of 1,3-diphenylallyl acetate with dimethyl malonate. The D-*fructo*-PyOx ligand provided mainly the (*R*)-enantiomer while the D-*psico*-configurated ligand gave the (*S*)-enantiomer with a lower enantiomeric excess.

## Introduction

The design of new chiral ligands for stereo-differentiating metal catalysts that enable asymmetric syntheses is still a highly active field of research in organic chemistry, for there is a continuously growing demand for enantiomeric pure building blocks for pharmaceuticals, agrochemicals or flavors. Carbohydrates are inexpensive and easy to obtain enantiomerically pure natural products and therefore, nearly ideal starting materials for ex-chiral pool syntheses. In addition, carbohydrates have significantly gained attention as ligands for metal complexes that enable asymmetric catalysis over the past decades, and an array of highly efficient privileged ligands for metal-catalyzed enantioselective syntheses have been derived from carbohydrates so far [1–6]. However, the enantioselective construction of C–C bonds, especially of tertiary carbon stereocenters, remains an ongoing challenge. Over the last decades though, transition metal-catalyzed reactions like the asymmetric allylic alkylation (Tsuji–Trost reaction) have evolved into one of the more powerful tools for synthesizing such tertiary stereocenters [7–8]. As a benchmark test for selectivity, the palladium-catalyzed asymmetric addition of dimethyl malonate to 1,3-diphenylallyl acetate was often used in the literature for testing the scope of carbohydrate derived ligands for this purpose [9–13]. For instance, Kunz and Gläser have demonstrated the stereo-differentiating potential of carbohydrate ligands in this type of reaction where their *gluco*-PHOX ligand, derived from glucosamine, resulted in a high enantiomeric excess of up to 98% [14].

**Figure 1:**
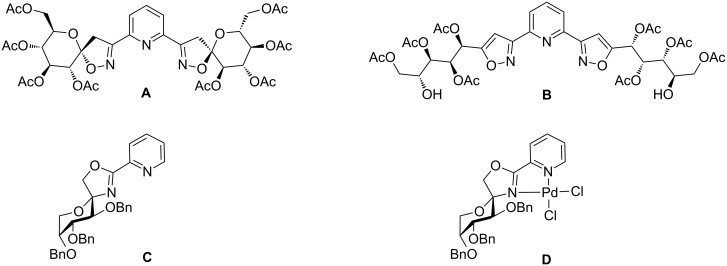
Pd-catalyzed cleavage of spiro-bis(isooxazoline) ligand **A** to isoxazole **B** and Pd-complex **D** prepared from spiro-oxazoline **C**.

Recently, Vidal et al. reported on a spiro-bis(isooxazoline) ligand **A** (Figure 1) [15] prepared via 1,3-dipolar cycloaddition of 2,6-pyridinedicarbonitrile *N,N*-dioxide to acetyl protected *exo*-glucal. The performance of ligand **A** in asymmetric catalysis was then tested in the palladium-catalyzed allylic addition of dimethyl malonate to 1,3-diphenylallyl acetate which, however, afforded the desired allylic substitution product only in traces. The authors attributed the inefficiency of ligand **A** to the cleavage of the spiro moiety resulting in the formation of thermodynamically more stable aromatic isoxazole **B** (Figure 1).

As part of our ongoing research program towards the design of carbohydrate derived ligands for metal-catalyzed asymmetric syntheses [16] we recently described the preparation of spiro-fused oxazoline ligands of type **C** from D-fructose [17]. We could demonstrate that our oxazoline ligands, in contrast to **A**, were able to form air and moisture-stable palladium complexes of type **D** upon reaction with Pd(cod)Cl_2_ (Figure 1). Single crystal X-ray diffraction of these Pd complexes revealed some interesting structural features in terms of ligand–metal bite angles and shielding of the palladium center by the carbohydrate scaffold from one specific side [17]. For example, we concluded from the crystallographic data that the orientation of the OBn group at C-3 in **D** could have a major impact on the “shielded” side of the metal center and thus, also on the stereoselectivity of the palladium-catalyzed allylic addition of dimethyl malonate to 1,3-diphenylallyl acetate. Encouraged by these aspects and in order to further our investigations in asymmetric catalysis with spiro-fused oxazoline ligands, we devised a new convenient synthetic strategy. Herein we present a new straightforward synthesis of spiro-fused D-*fructo*- and D-*psico*-configurated PyOx ligands and their application in palladium-catalyzed asymmetric alkylation.

## Results and Discussion

**Scheme 1:**
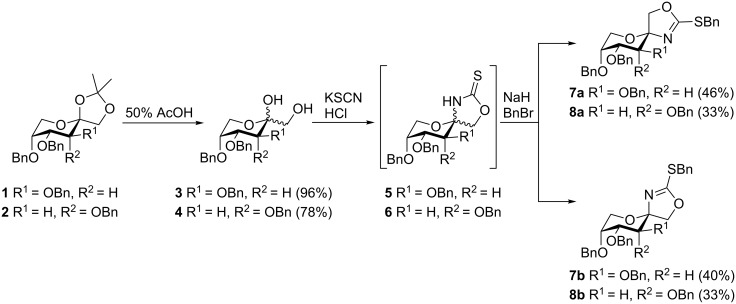
Synthesis of 2-benzylsulfanyl-1,3-oxazolines **7** and **8**.

We started our synthesis from readily available 3,4,5-tri-*O*-benzyl-1,2-*O*-isopropylidene-β-D-fructopyranose (**1**) [18] and 3,4,5-tri-*O*-benzyl-1,2-*O*-isopropylidene-β-D-psicopyranose (**2**) [19], respectively (Scheme 1). Deprotection of the isopropylidene group under acidic conditions gave the corresponding diols **3** [20] and **4** as anomeric mixtures. Condensation of the latter with thiocyanic acid in a Ritter-type reaction according to a slightly modified procedure described by Tatibouët et al. [21] gave an anomeric mixture of 1,3-oxazolidin-2-thiones **5** and **6**, respectively. The anomers of **5** and **6** could not be separated by standard column chromatography though. Thiocarbamate **5** was previously mentioned in the literature [21], but due to the fast anomerisation and the relative instability of 1,3-oxazolidin-2-thiones it was never characterized. However, in our hands, anomers could easily be separated by chromatography after benzylation of **5** and **6** with BnBr and NaH to give the corresponding benzylated sulfanyloxazolines **7** and **8** which were air and moisture stable (see Supporting Information File 1 for full experimental details). Nevertheless, it should be noted that these compounds slowly start to decompose after 2 weeks at −28 °C under an atmosphere of nitrogen.

**Scheme 2:**
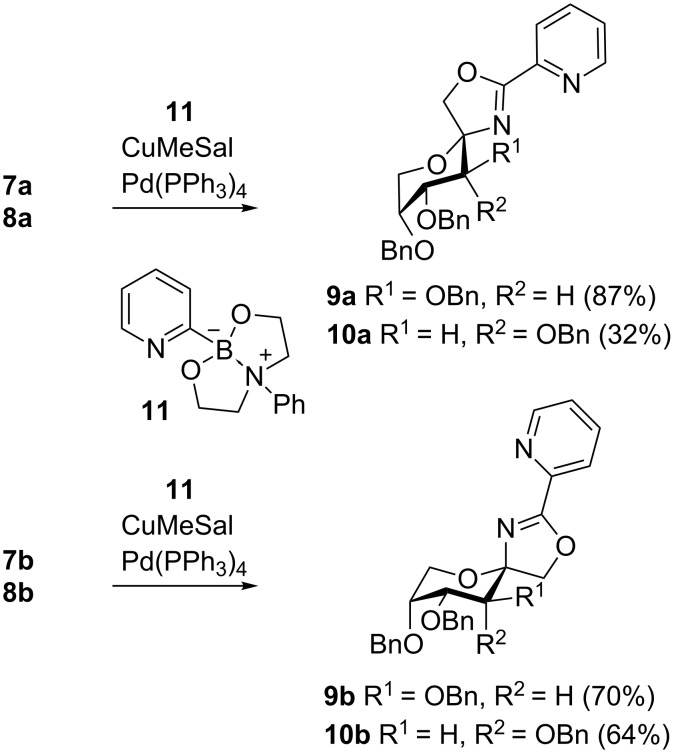
Pd-catalyzed cross coupling of benzylsulfanyloxazolines **7** and **8**.

Next, benzylsulfanyloxazolines **7** and **8** were subjected to copper-assisted palladium-catalyzed Suzuki–Miyaura-type cross coupling with commercially available 2-pyridineboronic acid *N*-phenyldiethanolamine ester **11** as boron source (Scheme 2) [22–24]. 2-Pyridineboronic acid **11** was chosen due to its increased nucleophilicity compared to other boron sources like pinacol boronic esters or MIDA boronates [25–26]. The cross coupling proceeded smoothly in THF and gave ligands **9** and **10** in good yields (Scheme 2). It is noteworthy that the low yield (32%) of **10a** is due to the instability of its spiro-fused oxazoline moiety during chromatography on silica gel but not to any unwanted side reactions during the cross coupling. Even small traces of HCl usually present in CDCl_3_ as impurity resulted in full decomposition of **10a** in seconds.

**Scheme 3:**
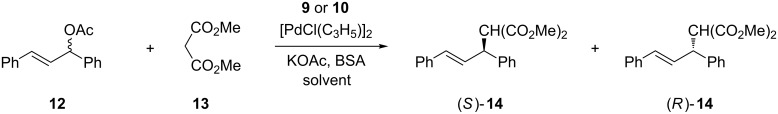
Palladium catalyzed allylic substitution.

With ligands **9** and **10** in hand, we moved on to the application in asymmetric catalysis. As a model system, the Pd-catalyzed allylic alkylation of dimethyl malonate (**13**) to *rac*-1,3-diphenylallyl acetate (**12**) was chosen (Scheme 3). As mentioned above, this reaction was often used as a benchmark for new chiral ligands and examined in great detail [9–14,27–28].

In all cases investigated here, the alkylated product **14** was isolated after purification by chromatography and its enantiomeric excess was determined via chiral HPLC using a Reprosil chiral-NR column. The absolute configuration was assigned by comparison of the optical rotation values with literature data [29] which are based on the chemical correlation method leading to (+)-(*S*)-2-phenylsuccinate [30] and by X-ray structure determination of (*R*,*E*)-3,5-diphenylpent-4-enyl camphor-10-sulfonate [31]. Thus, a positive optical rotation value refers to the (*R*)-enantiomer, whereas a negative value belongs to the (*S*)-enantiomer. In addition, the absolute configuration was independently determined by ^1^H NMR in the presence of the optically active NMR shift reagent (+)-Eu(hfc)_3_ [29]. All synthesized ligands were active precatalysts for the allylic substitution, as can be seen in Table 1.

**Table 1:** Pd-catalyzed allylic alkylation using ligands **9** and **10**.

Entry	Ligand	Solvent	Yield^a^	ee^b^

1	**9a**	CH_2_Cl_2_	74%	67% (*R*)
2	**9b**	CH_2_Cl_2_	80%	9% (*R*)
3	**9a**	MePh	68%	76% (*R*)
4	**9a**	MeCN	80%	59% (*R*)
5^c^	**9a**	CH_2_Cl_2_	80%	71% (*R*)
6^c^	**9a**	MePh	62%	88% (*R*)
7^d^	**9a**	MePh	43%	93% (*R*)
8	**10a**	MePh	traces	n.d.
9	**10a**	CH_2_Cl_2_	traces	n.d.
10^e^	**10a**	Cl(CH_2_)_2_Cl	43%	59% (*S*)
11	**10b**	MePh	56%	9% (*S*)

^a^Isolated yield after chromatography. ^b^Determined by chiral HPLC. ^c^*T* = 0 °C. ^d^*T* = −20 °C. ^e^*T* = 50 °C.

The asymmetric allylic alkylation was carried out in the presence of 5 mol % [PdCl(C_3_H_5_)]_2_ and 11 mol % chiral ligands **9** and **10**, respectively. The D-*fructo*-configurated ligands **9a** and **9b** showed preparative yields for (*R*)-**14** in the range of 74–80% (Table 1, entries 1 and 2). The α-anomer **9a** showed a significant higher selectivity (67% ee) than the β-anomer **9b** (9% ee). In order to investigate the solvent and temperature effects of the reaction we conducted further studies with the more selective ligand **9a**. While the reaction proceeded smoothly in all tested solvents, the enantiomeric excess increased in toluene (76% ee, Table 1, entry 3), whereas acetonitrile had a negative effect on selectivity (Table 1, entry 4). Lowering the temperature increased the selectivity of the reaction as well (Table 1, entries 5–7). Conducting the reaction at a temperature of −20 °C yielded (*R*)-**14** with an enantiomeric excess of 93%. Surprisingly, when the D-*psico*-configurated ligand **10a** was used in toluene or methylene chloride under conditions identical to those used with ligand **9a**, only traces of the alkylated product **14** could be obtained. To our delight, however, ligand **10a** was active in 1,2-dichloroethane at 50 °C and gave the opposite enantiomer (*S*)-**14** with an enantiomeric excess of 59% (Table 1, entry 10). Similar to ligand **9b**, the β-configurated D-*psico*-ligand **10b** leads to a somewhat lower enantiomeric excess of (*S*)-**14** of 9% (Table 1, entry 11).

**Scheme 4:**
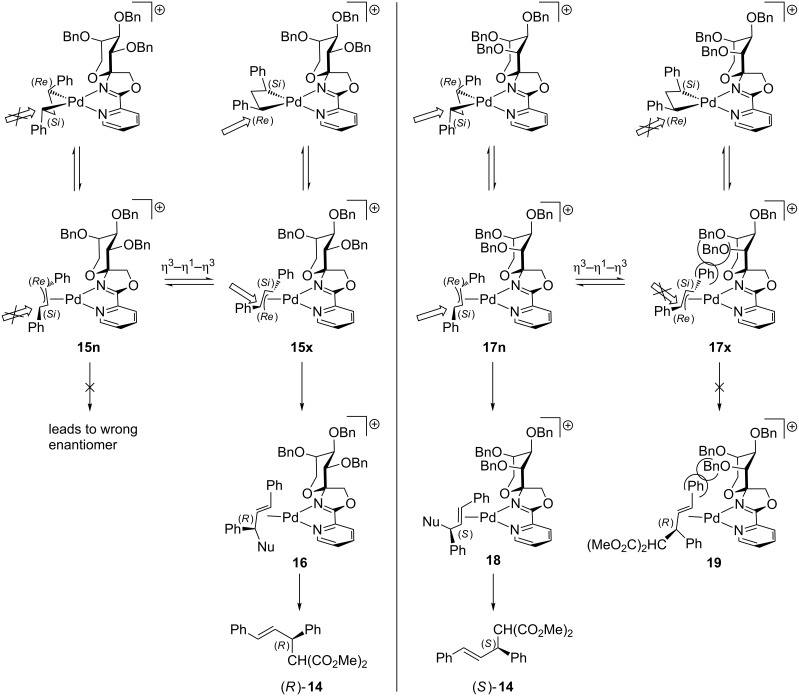
Proposed transition state of allylic substitution.

The stereoselectivity of the Pd-catalyzed allylic substitution can be explained via a model for the proposed transition state (Scheme 4). As a consequence of the spiro-fused carbohydrate moiety at the oxazoline ring, *exo* (**15x** and **17x**) and *endo* (**15n** and **17n**) diastereomers of the palladium complexes can be distinguished. It is also reasonable to assume that *exo*/*endo* isomers **15** and **17** undergo fast allyl rotation via a η^3^–η^1^–η^3^ isomerization mechanism and thus, exist in a dynamic equilibrium which is approximately ten to hundred times faster than alkylation [7]. Therefore, four reaction pathways are possible, but only two lead to the observed stereoselectivties. We assume, that the nucleophilic attack occurs at the allyl terminus *trans* to the oxazoline ring, which is in accordance with previously reported findings in allylic substitution using PyOx ligands [32–33]. If the nucleophile attacks from the (*Si*)-face in the *fructo*-configurated complex **15n** product **14** must have the (*S*)-configuration which is, however, contrary to the observed steroselectivity. Therefore, we suggest **15x** to be the preferred isomer for attack by the nucleophile from the (*Re*)-face which leads to the η^2^-complex **16** with (*R*)-configuration.

In the case of D-*psico*-ligand **10a**, the (*S*)-enantiomer was found to be the major enantiomer. That leads to the assumption that the *endo* complex **17n** is the predominant species attacked by the nucleophile from the (*Si*)-face which results in η^2^-complex **18** exhibiting (*S*)-configuration. Although the *exo* complexes are assumed to be thermodynamically more stable than the *endo* isomers, complex **17x** is disfavoured, because of the steric repulsion of the phenyl group in the allylic substrate and the OBn group at C-3 of the D-psicose moiety. This assumption also explains our observation, that the D-*psico*-configurated ligand **10a** requires higher temperatures for formation of the *endo* complex **17n** and thus, results in a slower reaction rate and in a lower enantioselectivity compared to ligand **9a**.

## Conclusion

In summary, we have synthesized four spiro-fused carbohydrate ligands in the D-*fructo*- and D*-psico*-series via a straightforward synthetic route. The key steps in our synthesis were a Ritter type condensation reaction of partially benzyl-protected D-fructose and D-psicose derivatives with thiocyanic acid to afford the corresponding 1,3-oxazolidine-2-thiones, and the cross coupling of the latter under modified Suzuki–Miyaura conditions. The prepared ligands were shown to be active precatalysts for the asymmetric allylic alkylation of 1,3-diphenylallyl acetate with dimethyl malonate. The D-*fructo*-configurated ligands provided the (*R*)-enantiomer with up to 93% ee, whereas the D-*psico*-configurated ligands gave the (*S*)-enantiomer in a somewhat lower selectivity (up to 59% ee). The stereochemical outcome of the reaction could be explained by a proposed transition state of the allylic substitution. Further insights into the reaction mechanism of the allylic substitution using similar PyOx ligands are currently under investigation and will be published elsewhere.

## Supporting Information

File 1 Experimental procedures, analytical data and copies of NMR spectra.
