# Supplementary material for: Spiro-fused carbohydrate oxazoline ligands: Synthesis and application as enantio-discrimination agents in asymmetric allylic alkylation
Source: Beilstein J Org Chem. 2016 Jan 29;12:166–71. doi: 10.3762/bjoc.12.18 (PMC4734355; doi:10.3762/bjoc.12.18)
Supplement: File 1 — Experimental procedures, analytical data and copies of NMR spectra. [file Beilstein_J_Org_Chem-12-166-s001.pdf]

Supporting Information

for

Spiro-fused carbohydrate oxazoline ligands: Synthesis and application as enantio-discrimination agents in asymmetric allylic alkylation

Jochen Kraft1, Martin Golkowski2 and Thomas Ziegler*1

Address: 1Institute of Organic Chemistry, University of Tuebingen, Auf der Morgenstelle 18, 72076 Tuebingen, Germany and 2Department of Pharmacology, University of Washington, 1959 NE Pacific St, Box 357280, Seattle, WA 98195, USA

Email: Thomas Ziegler* - thomas.ziegler@uni-tuebingen.de

*Corresponding author

Experimental procedures, analytical data and copies of NMR spectra

Content

Experimental procedures	S3–S12
References	S13
NMR spectra	S14–S20

Experimental procedures

General remarks

All solvents were dried according to standard methods, distilled and stored over molecular sieves 3 Å under an atmosphere of nitrogen prior to their use. Petroleum ether (PE) refers to the fraction boiling in the range 60–90 °C. All non-aqueous reactions were performed in oven-dried glassware under an atmosphere of N_2 unless stated otherwise. NMR spectra were recorded on a Bruker Avance 400 spectrometer and were calibrated for the respective solvent signal (^{1}H-$CDCl_3$: 7.26 ppm; ^{13}C-$CDCl_3$: 77.16 ppm). NMR signals were numbered in accordance with carbohydrate nomenclature for the names of the compounds. ESI-HRMS were measured on a Bruker Daltonics MAXIS 4G spectrometer. ESI-spectra were recorded on a Bruker Esquire 3000 Plus spectrometer. Elemental analyses were performed on a HEKAtech Euro 3000 CHN analyzer. IR spectra were recorded on a Bruker Tensor 27 spectrometer. Optical rotations were measured with a Perkin-Elmer Polarimeter 341 in a 10 cm cuvette at 20 °C. Melting points were determined with a Büchi Melting Point M-560 apparatus. Reactions were monitored by TLC on Polygram Sil G/UV silica gel plates from Machery&Nagel. Detection of spots was effected by charring with H_2SO_4 (5% in EtOH), staining by spraying the plates with an alkaline aqueous solution of potassium permanganate or by inspection of the TLC plates under UV light. Preparative chromatography was performed on silica gel (0.032–0.063 mm) from Machery&Nagel with different mixtures of solvents as eluent. The enantiomeric excess of **14** was determined by chiral HPLC on a Sykam S 1121 HPLC system equipped with a Reprosil Chiral-NR column (*n*-hexane:2-propanol, 90:10; flow rate 1.6 mL/min): t_R = 6.8 min for (*R*)-**14**, t_R = 8.6 min for (*S*)-**14**. All yields are isolated yields determined after purification of the product either by silica gel column chromatography or crystallization and were not optimized unless noted otherwise.

3,4,5-Tri-*O*-benzyl-D-fructopyranose (3)

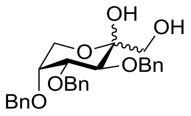

3

3,4,5-Tri-*O*-benzyl-1,2-*O*-isopropylidene-D-fructopyranose (**1**) [1] (0.94 g, 1.91 mmol) was dissolved in 50% aqueous acetic acid (20 mL), and the solution was refluxed for 3 h. After cooling the solution to room temperature, the resulting suspension was evaporated in vacuo and coevaporated with toluene. Chromatography of the residue (PE:EtOAc, 1:1) afforded **3** (0.83 g, 96%) as colorless oil. Spectroscopic data were in accordance with the literature [2].

3,4,5-Tri-*O*-benzyl-D-psicopyranose (4)

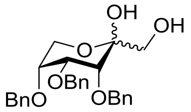

4

A suspension of 3,4,5-tri-*O*-benzyl-1,2-*O*-isopropylidene-D-psicopyranose (**2**) [3] (0.98 g, 2.00 mmol) in 50% aqueous acetic acid (20 mL) was refluxed for 3 h. The reaction mixture was cooled to room temperature concentrated in vacuo and coevaporated with toluene. Chromatography of the residue (PE:EtOAc, 2:1) and crystallization from *n*-hexane/EtOAc afforded **4** (0.70 g, 78%) as colorless needles. Mp 101 °C (*n*-hexane/EtOAc). R_f = 0.40 (PE:EtOAc, 1:1). ^{1}H NMR ($CDCl_3$): δ 7.40-7.30 (m, 15H, phenyl-H), 5.71 (bs, 1H, OH), 4.92 (d, *J* = 11.5 Hz, 1H, OCH_2Ph), 4.78 (d, *J* = 11.5 Hz, 1H, OCH_2Ph), 4.60-4.52 (m, 4H, OCH_2Ph), 4.29 (bs, 1H, H-5), 4.06 (t, *J* = 11.0 Hz, 1H, H-6a), 3.77-3.69 (m, 2H, H-1a, H-6b), 3.51-3.45 (m, 3H, H-1b, H-3, H-4), 1.76 (bs, 1H, OH). ^{13}C NMR ($CDCl_3$): δ 138.0, 137.8, 137.7, 128.7, 128.7, 128.6, 128.3, 128.2, 128.1, 128.1, 127.6 (C-Ar), 97.8 (C-2), 75.7 (C-5), 75.3 (OCH_2Ph), 75.0, 73.2 (C-3, C-4), 71.7, 71.6 (OCH_2Ph), 64.8 (C-1), 57.6 (C-6). MS

(ESI): *m/z* 489.2 $[M+K]^+$, 473.3 $[M+Na]^+$. Anal. calcd for $C_{27}H_{30}O_6$ (450.5): C, 71.98; H, 6.71; found: C, 72.00; H, 6.72.

(5*S*,8*R*,9*R*,10*S*)-8,9,10-Tris(benzyloxy)-2-benzylthio-3,6-dioxa-1-azaspiro[4.5]dec-1-ene (7a) and (5*R*,8*R*,9*R*,10*S*)-8,9,10-Tris(benzyloxy)-2-benzylthio-3,6-dioxa-1-azaspiro[4.5]-dec-1-ene (7b)

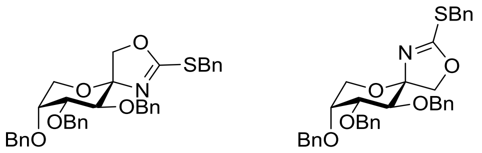

7a **7b**

To a stirred solution of 3,4,5-tri-*O*-benzyl-D-fructopyranose (**3**) [2] (0.76 g, 1.69 mmol) in 50% aqueous 1,2-dimethoxyethane (20 mL) was added KSCN (0.41 g, 4.22 mmol) and conc. aqueous HCl solution (37%, 0.34 mL, 4.22 mmol). The resulting mixture was stirred at 90 °C for 72 h, evaporated in vacuo and re-dissolved in EtOAc (30 mL). The organic phase was washed with satd. aqueous $NaHCO_3$ solution and H_2O, dried over Na_2SO_4, filtered and concentrated. The obtained colorless foam was dissolved in dry DMF (15 mL) and cooled to 0 °C. Benzyl bromide (0.30 mL, 2.53 mmol) and NaH (0.08 g, 2.02 mmol; 60% dispersion in mineral oil) were added and the resulting solution was stirred for 1 h at 0 °C and an additional 12 h at room temperature. The reaction mixture was quenched by addition of MeOH (5 mL) and evaporated to dryness. The residue was re-dissolved in EtOAc (30 mL), washed with H_2O and brine, dried over Na_2SO_4, filtered and concentrated. Chromatography of the residue (toluene:EtOAc, 35:1 +2.5% Et_3N) afforded two fractions. Eluted first was **7b** (0.39 g, 40%) which was obtained as a colorless oil. $[\alpha]_D^{20}$ -100.9 (c 1.00, $CHCl_3$). R_f = 0.38 (toluene-EtOAc, 30:1 +2.5% Et_3N). ^1H NMR ($CDCl_3$): δ 7.44-7.24 (m, 20H, phenyl-H), 5.06 (d, *J* = 11.9 Hz, 1H, OCH_2Ph), 4.82-4.65 (m, 5H, OCH_2Ph), 4.28 (q, *J* = 12.3 Hz, 2H, SCH_2Ph),

4.20-4.15 (m, 3H, H-4, H-1a, H-1b), 4.04-4.00 (m, 2H, H-6a, H-3), 3.88 (bs, 1H, H-5), 3.81 (dd, $J_{6b,6a}$ = 12.5 Hz, $J_{6b,5}$ = 1.9 Hz, 1H, H-6b). ^{13}C NMR ($CDCl_3$): δ 169.5 (OCN), 138.6, 138.5, 138.4, 137.1 129.0, 128.6, 128.5, 128.5, 128.4, 128.0, 127.8, 127.7, 127.7, 127.6, 127.5 (C-Ar), 102.6 (C-2), 80.4 (C-4), 77.3 (C-3), 76.5 (C-1), 75.0 (OCH_2Ph), 73.8 (C-5), 72.2, 71.8 (OCH_2Ph), 62.9 (C-6), 36.3 (SCH_2Ph). MS (ESI): *m/z* 620 $[M+K]^+$, 604 $[M+Na]^+$, 582 $[M+H]^+$. HRMS (ESI-TOF): *m/z* calcd for $C_{35}H_{36}NO_5S$ $[M+H]^+$: 582.23087; found: 582.23116.

Eluted next was **7a** (0.45 g, 46%) which was obtained as colorless oil. $[\alpha]_D^{20}$ +3.6 (c 1.00, $CHCl_3$). R_f = 0.32 (toluene-EtOAc, 30:1 +2.5% Et_3N). ^{1}H NMR ($CDCl_3$): δ 7.43-7.17 (m, 20H, phenyl-H), 4.81 (d, J = 12.6 Hz, 1H, OCH_2Ph), 4.69-4.64 (m, 4H, OCH_2Ph, H-1a, H-1b), 4.49 (dd, J = 9.8 Hz, J = 8.7 Hz, 2H, OCH_2Ph), 4.39-4.23 (m, 3H, SCH_2Ph, OCH_2Ph), 4.19 (d, $J_{3,4}$ = 9.3 Hz, 1H, H-3), 4.10 (dd, $J_{6a,6b}$ = 12.8 Hz, $J_{6a,5}$ = 3.6 Hz, 1H, H-6a), 3.76-3.75 (m, 1H, H-5), 3.44 (dd, $J_{4,3}$ = 9.3 Hz, $J_{4,5}$ = 3.1 Hz, 1H, H-4), 3.34 (dd, $J_{6b,6a}$ = 12.9 Hz, $J_{6b,5}$ = 1.4 Hz, 1H, H-6b). ^{13}C NMR ($CDCl_3$): δ 170.7 (OCN), 138.5, 138.4, 138.2, 136.5, 129.3, 128.7, 128.6, 128.5, 128.5, 128.4, 128.1, 128.1, 127.8, 127.7, 127.7, 127.7, 127.6 (C-Ar), 103.6 (C-2), 79.0 (C-3), 78.5 (C-4), 75.3, 73.1 (OCH_2Ph), 72.3 (C-5), 72.3 (C-1), 71.3 (OCH_2Ph), 62.1 (C-6), 36.6 (SCH_2Ph). HRMS (ESI-TOF): *m/z* calcd for $C_{35}H_{36}NO_5S$ $[M+H]^+$: 582.23087; found: 582.23130. Anal. Calcd for $C_{35}H_{35}NO_5S$ (581.7): C, 72.26; H, 6.06; N, 2.41; S, 5.51; found.: C, 72.13; H, 6.13; N, 2.44; S, 5.32.

(5*S*,8*R*,9*R*,10*R*)-8,9,10-Tris(benzyloxy)-2-benzylthio-3,6-dioxa-1-azaspiro[4.5]dec-1-ene (8a) and (5*R*,8*R*,9*R*,10*R*)-8,9,10-Tris(benzyloxy)-2-benzylthio-3,6-dioxa-1-azaspiro[4.5]-dec-1-ene (8b)

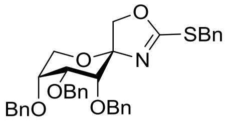

8a

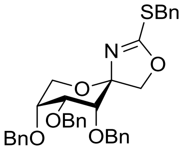

8b

To a stirred solution of 3,4,5-tri-*O*-benzyl-D-psicopyranose (**4**, 1.60 g, 3.60 mmol) in 50% aqueous 1,2-dimethoxyethane (50 mL) was added KSCN (0.88 g, 9.10 mmol) and conc. aqueous HCl-solution (37%, 0.75 mL, 9.10 mmol). The resulting mixture was stirred at 90 °C for 72 h, evaporated in vacuo and re-dissolved in EtOAc (50 mL). The organic phase was washed with satd. aqueous $NaHCO_3$-solution and H_2O, dried over Na_2SO_4, filtered and concentrated. The obtained colorless foam was dissolved in dry DMF (30 mL) and cooled to 0 °C. Benzyl bromide (0.65 mL, 5.43 mmol) and NaH (0.17 g, 4.34 mmol; 60% dispersion in mineral oil) were added and the resulting solution was stirred for 1 h at 0 °C followed by room temperature for 12 h. The reaction mixture was quenched by the addition of MeOH (10 mL) and evaporated to dryness. The residue was re-dissolved in EtOAc (50 mL), washed with H_2O and brine, dried over Na_2SO_4, filtered and concentrated. Chromatography of the residue (toluene:EtOAc, 35:1 +2.5% Et_3N) afforded two fractions. Eluted first was **8a** (0.70 g, 33%) which was obtained as a colorless oil. $[\alpha]_D^{20}$ +68.6 (c 1.00, $CHCl_3$). R_f = 0.53 (toluene:EtOAc, 35:1 +2.5% Et_3N). ^{1}H NMR ($CDCl_3$): δ 7.53-7.22 (m, 20H, phenyl-H), 4.97 (q, J = 13.9 Hz, 2H, OCH_2Ph), 4.75-4.65 (m, 3H, OCH_2Ph), 4.52-4.36 (m, 5H, OCH_2Ph, SCH_2Ph, H-6a, H-4), 4.13 (d, $J_{1a,1b}$ = 9.0 Hz, 1H, H-1a), 4.00 (d, $J_{1b,1a}$ = 9.0 Hz, 1H, H-1b), 3.70-3.66 (m, 1H, H-6b), 3.62-3.58 (m, 1H, H-5), 3.32 (d, $J_{3,4}$ = 2.8 Hz, 1H, H-3). ^{13}C NMR ($CDCl_3$): δ 169.4 (OCN), 139.7, 138.3, 137.5, 129.2, 128.6, 128.6, 128.5, 128.1, 128.0, 127.9, 127.6, 127.5, 127.4, 127.1 (C-Ar), 100.4 (C-2), 77.9 (C-3), 76.9 (C-1), 75.5 (C-5), 73.4 (OCH_2Ph), 72.0 (C-4), 71.2, 70.9 (OCH_2Ph), 60.0 (C-6), 36.7 (SCH_2Ph). MS (ESI): *m/z* 620.1

[M+K]$^+$, 604.2 [M+Na]$^+$, 582.2 [M+H]$^+$. HRMS (ESI-TOF): *m/z* calcd for $C_{33}H_{36}NO_5S$ [M+H]$^+$: 582.23087; found: 582.23156.

Eluted next was **8b** (0.70 g, 33%) which was obtained as colorless oil. $[\alpha]_D^{20}$ -2.5 (c 1.00, $CHCl_3$). R_f = 0.29 (toluene:EtOAc, 35:1 +2.5% Et_3N). ^{1}H NMR ($CDCl_3$): δ 7.30-7.15 (m, 20H, phenyl-H), 4.74-4.42 (m, 7H, OCH_2Ph, H-1a, H-1b), 4.35-4.29 (m, 2H, OCH_2Ph, SCH_2Ph), 4.20 (d, *J* = 13.3 Hz, 1H, SCH_2Ph), 4.02 (bs, 1H, H-4), 3.78-3.65 (m, 3H, H-6a, H-6b, H-3), 3.55-3.50 (m, 1H, H-5). ^{13}C NMR ($CDCl_3$): δ 138.8, 138.4, 138.0, 136.2, 129.1, 128.8, 128.6, 128.5, 128.4, 128.0, 127.9, 127.8, 127.7, 127.7 (C-Ar), 101.0 (C-2), 78.3 (C-3), 77.4 (C-1), 75.3 (C-4), 74.5 (C-5), 74.3, 73.0, 71.5 (OCH_2Ph), 60.6 (C-6), 36.6 (SCH_2Ph). MS (ESI): *m/z* 620.1 [M+K]$^+$, 604.2 [M+Na]$^+$, 582.2 [M+H]$^+$. Anal. calcd for $C_{35}H_{35}NO_5S$ (581.7): C, 72.26; H, 6.06; N, 2.41; found: C, 72.28; H, 6.11; N, 2.39.

General procedure for the copper-assisted palladium-catalyzed cross coupling

To a stirred solution of 2-benzylsulfanyl-1,3-oxazolines **7** or **8** (1 equiv) in dry THF (15 mL) was added at room temperature in the following order: 2-pyridineboronic acid *N*-phenyldiethanolamine ester **11** (2.2 equiv), copper (I) 3-methylsalicylate (CuMeSal) (2.2 equiv) and $Pd(PPh_3)_4$ (5 mol %). Stirring of the mixture was continued for 14 h at 70 °C. The mixture was cooled to room temperature, diluted with satd. aqueous Na_2CO_3 solution (5 mL) and extracted with EtOAc (3 × 10 mL). The combined organic phases were washed with H_2O and brine, dried over Na_2SO_4, filtered and concentrated. Chromatography of the residue gave the cross-coupled compounds **9** and **10**.

(5*S*,8*R*,9*R*,10*S*)-8,9,10-Tris(benzyloxy)-2-(pyridin-2-yl)-3,6-dioxa-1-azaspiro[4.5]dec-1-ene (9a)

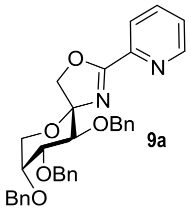

9a

Treatment of **7a** (100 mg, 0.17 mmol) with 2-pyridineboronic acid *N*-phenyldiethanolamine ester **11** (101 mg, 0.38 mmol), CuMeSal (81 mg, 0.38 mmol) and Pd(PPh_3)$_4$ (10 mg, 0.01 mmol, 5 mol %) according to the general procedure followed by column chromatography (toluene:EtOAc, 1:1 +2.5% Et_3N) and recrystallization from *n*-hexane/EtOAc gave **9a** (80 mg, 87%) as colorless needles. Spectroscopic data were in accordance with previously published data [4].

(5*R*,8*R*,9*R*,10*S*)-8,9,10-Tris(benzyloxy)-2-(pyridin-2-yl)-3,6-dioxa-1-azaspiro[4.5]dec-1-ene (9b)

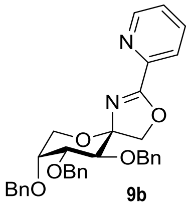

9b

Treatment of **7b** (140 mg, 0.24 mmol) with 2-pyridineboronic acid *N*-phenyldiethanolamine ester **11** (142 mg, 0.53 mmol), CuMeSal (114 mg, 0.53 mmol) and Pd(PPh_3)$_4$ (14 mg, 0.01 mmol, 5 mol%) according to the general procedure followed by column chromatography (toluene:EtOAc, 2:1 +2.5% Et_3N) and recrystallization from *n*-hexane/EtOAc gave **9b** (90 mg, 70%) as colorless needles. Spectroscopic data were in accordance with previously published data [4].

(5*S*,8*R*,9*R*,10*R*)-8,9,10-Tris(benzyloxy)-2-(pyridin-2-yl)-3,6-dioxa-1-azaspiro[4.5]dec-1-ene (10a)

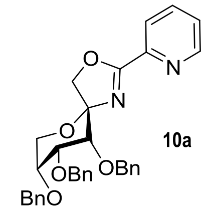

10a

Treatment of **8a** (0.54 g, 0.93 mmol) with 2-pyridineboronic acid *N*-phenyldiethanolamine ester **11** (0.55 g, 2.04 mmol), CuMeSal (0.44 g, 2.04 mmol) and $Pd(PPh_3)_4$ (0.05 g, 0.05 mmol) according to the general procedure followed by column chromatography (toluene:EtOAc, 35:1 +2.5% Et_3N) afforded **10a** (0.16 g, 32%) as a pale yellow oil. $[\alpha]_D^{20}$ +82.7 (c 1.00, $CHCl_3$). R_f = 0.20 (toluene:EtOAc, 35:1 +2.5% Et_3N). ^1H NMR ($CDCl_3$): δ 8.63 (d, *J* = 4.2 Hz, 1H, pyridine-H), 8.20 (d, *J* = 7.9 Hz, 1H, pyridine-H), 7.66 (td, *J* = 3.9 Hz, *J* = 1.7 Hz, 1H, pyridine-H), 7.47 (d, *J* = 6.8 Hz, 1H, pyridine-H), 7.33-7.15 (m, 15H, phenyl-H), 4.88 (q, *J* = 13.3 Hz, 2H, OCH_2Ph), 4.59-4.45 (m, 4H, OCH_2Ph, H-6a), 4.31-4.29 (m, 2H, OCH_2Ph, H-4), 4.14 (d, *J* = 9.5 Hz, 1H, H-1a), 4.03 (d, *J* = 9.5 Hz, 1H, H-1b), 3.61 (dd, $J_{6b,6a}$ = 10.5 Hz, $J_{6b,5}$ = 4.1 Hz, 1H, H-6b), 3.55-3.50 (m, 1H, H-5), 3.26 (d, $J_{3,4}$ = 2.7 Hz, 1H, H-3). ^{13}C NMR ($CDCl_3$): δ 165.2 (OCN), 149.5, 147.1, 139.8, 138.3, 137.2, 136.5, 128.6, 128.5, 128.3, 128.1, 128.1, 127.8, 127.5, 127.0, 125.8, 125.4 (C-Ar), 101.3 (C-2), 78.0 (C-3), 75.6 (C-1), 75.4 (C-5), 73.5 (OCH_2Ph), 72.2 (C-4), 71.2, 70.9 (OCH_2Ph), 60.2 (C-6). MS (ESI): *m/z* 559.2 $[M+Na]^+$, 537.2 $[M+H]^+$. HRMS (ESI-TOF): *m/z* calcd for $C_{33}H_{33}N_2O_5$ $[M+H]^+$: 537.23840; found: 537.23863.

(5*R*,8*R*,9*R*,10*R*)-8,9,10-Tris(benzyloxy)-2-(pyridin-2-yl)-3,6-dioxa-1-azaspiro[4.5]dec-1-ene (10b)

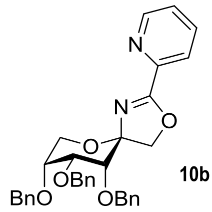

10b

Treatment of **8b** (0.22 g, 0.38 mmol) with 2-pyridineboronic acid *N*-phenyldiethanolamine ester **11** (0.22 g, 0.83 mmol), CuMeSal (0.18 g, 0.83 mmol) and $Pd(PPh_3)_4$ (0.02 g, 0.02 mmol) according to the general procedure followed by column chromatography (toluene:EtOAc, 2:1 +2.5% Et_3N) gave **10b** (0.13 g, 64%) as a colorless oil. $[\alpha]_D^{20}$ -73.6 (c 1.00, $CHCl_3$). R_f = 0.31 (toluene:EtOAc, 2:1 +2.5% Et_3N). ^{1}H NMR ($CDCl_3$): δ 8.75 (ddd, *J* = 4.7 Hz, *J* = 1.5 Hz, *J* = 0.8 Hz, 1H, pyridine-H), 8.18 (d, *J* = 7.9 Hz, 1H, pyridine-H), 7.80 (td, *J* = 3.9 Hz, *J* = 1.7 Hz, 1H, pyridine-H), 7.45-7.25 (m, 16H, pyridine-H, phenyl-H), 4.88-4.54 (m, 8H, OCH_2Ph, H-1a, H-1b), 4.20 (bs, 1H, H-4), 3.96-3.84 (m, 3H, H-3, H-6a, H-6b), 3.67 (ddd, *J* = 9.7 Hz, *J* = 4.8 Hz, *J* = 2.4 Hz, 1H, H-5). ^{13}C NMR ($CDCl_3$): δ 166.8 (OCN), 149.9, 138.9 138.4, 138.1, 136.8, 128.6, 128.4, 127.9, 127.9, 127.8, 127.7, 127.6, 126.2, 124.8 (C-Ar), 102.2 (C-2), 78.5 (C-3), 75.3 (C-4), 74.7 (C-5), 74.2, 72.8, 71.6 (OCH_2Ph, C-1), 60.3 (C-6). HRMS (ESI-TOF): *m/z* calcd for $C_{33}H_{33}N_2O_5$ $[M+H]^+$: 537.23840; found: 537.23881.

General procedure for the addition of dimethyl malonate to 1,3-diphenylallyl acetate

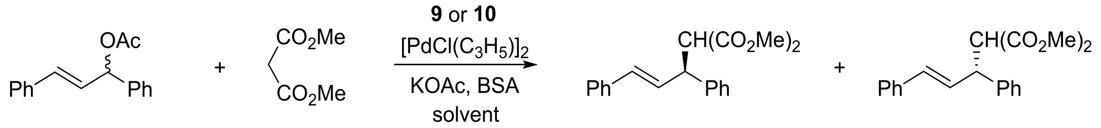

A solution of ligand **9** or **10** (6 mg, 0.011 mmol, 11 mol % with respect to acetate **12**) and $[PdCl(C_3H_5)]_2$ (1.8 mg, 0.005 mmol, 5 mol %) in dry toluene (1 mL) was stirred for 30 min at room temperature. Racemic (*E*)-1,3-diphenylallyl acetate (**12**, 25 mg, 0.1 mmol), dimethyl

malonate (**13**, 34 µL, 0.3 mmol), *N*,*O*-bis(trimethylsilyl)acetamide (BSA) (73 µL, 0.3 mmol) and KOAc (0.5 mg, 0.005 mmol) were added and stirring was continued for 12 h at room temperature. The reaction mixture was diluted with Et_2O (3 mL) and 5% aqueous NH_4Cl solution (2 mL) and the aqueous layer was extracted with Et_2O (3 × 5 mL). The combined organic phases were washed with water and brine, dried over Na_2SO_4, filtered and concentrated. Chromatography of the residue (PE:EtOAc, 6:1) gave **14** as a colorless oil. Spectroscopic data are in correspondence with literature [5].

References

1. Kaji, E.; Kurimoto, E.; Saiga, R.; Matsuura, A.; Harada, K.; Nishino, T. *Heterocycles* **2005**, *66*, 453-468.

2. Campbell, M. M.; Heffernan, G. D.; Lewis, T. *Carbohydr. Res.* **1994**, *251*, 243-250.

3. Nakajima, N.; Matsumoto, M.; Kirihara, M.; Hashimoto, M.; Katoh, T.; Terashima, S. *Tetrahedron* **1996**, *52*, 1177-1194.

4. Kraft, J.; Ziegler, T. *Carbohydr. Res.* **2015**, *411*, 56-63.

5. Leutenegger, U.; Umbricht, G.; Fahrni, C.; von Matt, P.; Pfaltz, A. *Tetrahedron* **1992**, *48*, 2143-2156.

NMR spectra

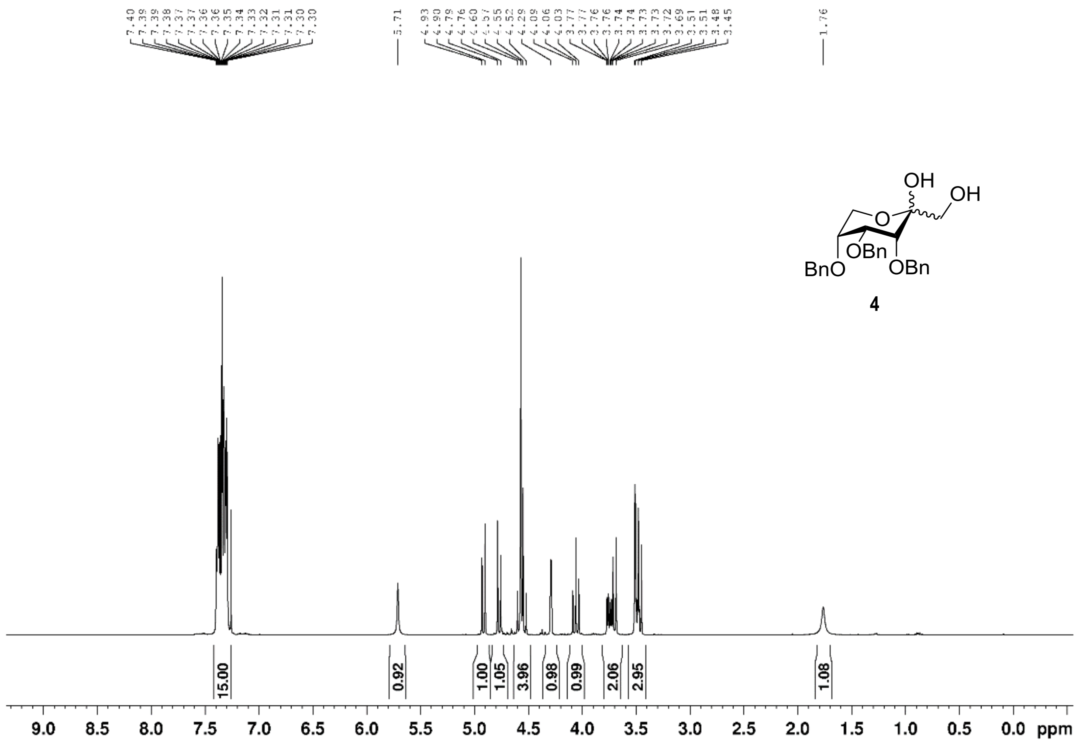
7.40
7.39
7.39
7.38
7.37
7.37
7.36
7.36
7.35
7.34
7.33
7.32
7.31
7.31
7.30
7.30
5.71
4.93
4.90
4.79
4.76
4.60
4.57
4.55
4.52
4.29
4.09
4.06
4.03
3.77
3.77
3.76
3.74
3.74
3.73
3.73
3.72
3.69
3.51
3.51
3.48
3.45
1.76
OH
OH
O
OBn
BnO
OBn
4
15.00
0.92
1.00
1.05
3.96
0.98
0.99
2.06
2.95
1.08
9.0
8.5
8.0
7.5
7.0
6.5
6.0
5.5
5.0
4.5
4.0
3.5
3.0
2.5
2.0
1.5
1.0
0.5
0.0 ppm

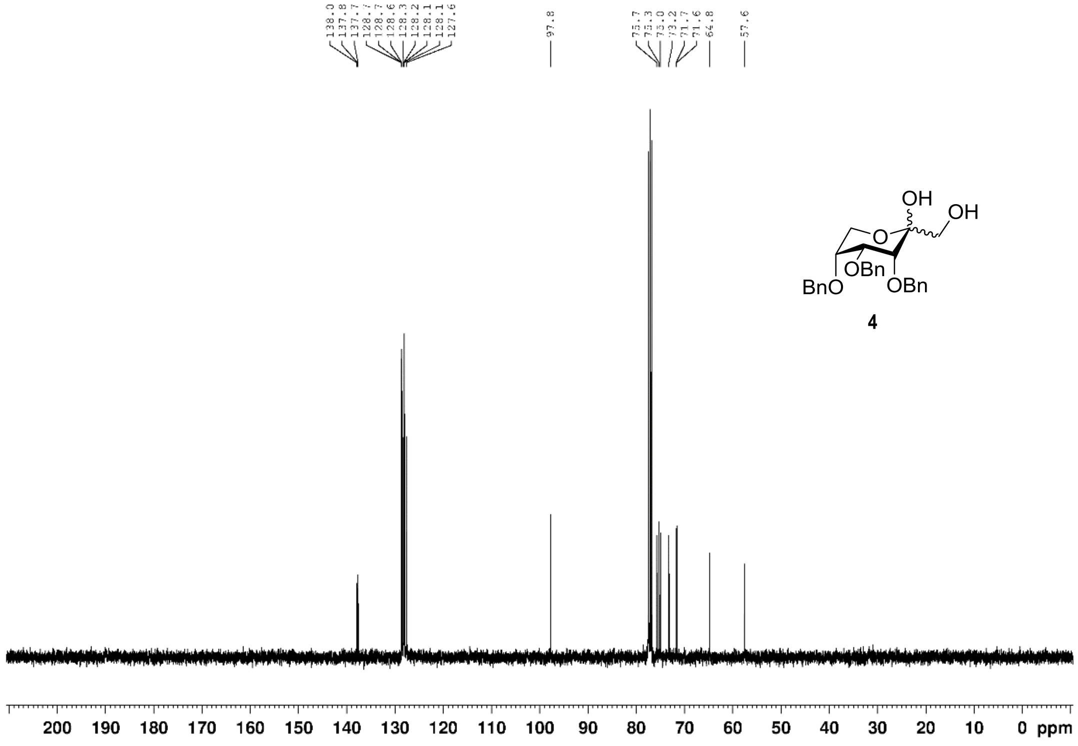
138.0
137.8
137.7
128.7
128.7
128.6
128.3
128.2
128.1
127.6
97.8
75.7
75.3
75.0
73.2
71.7
71.6
64.8
57.6
OH
OH
O
OBn
BnO
OBn
4
200
190
180
170
160
150
140
130
120
110
100
90
80
70
60
50
40
30
20
10
0 ppm

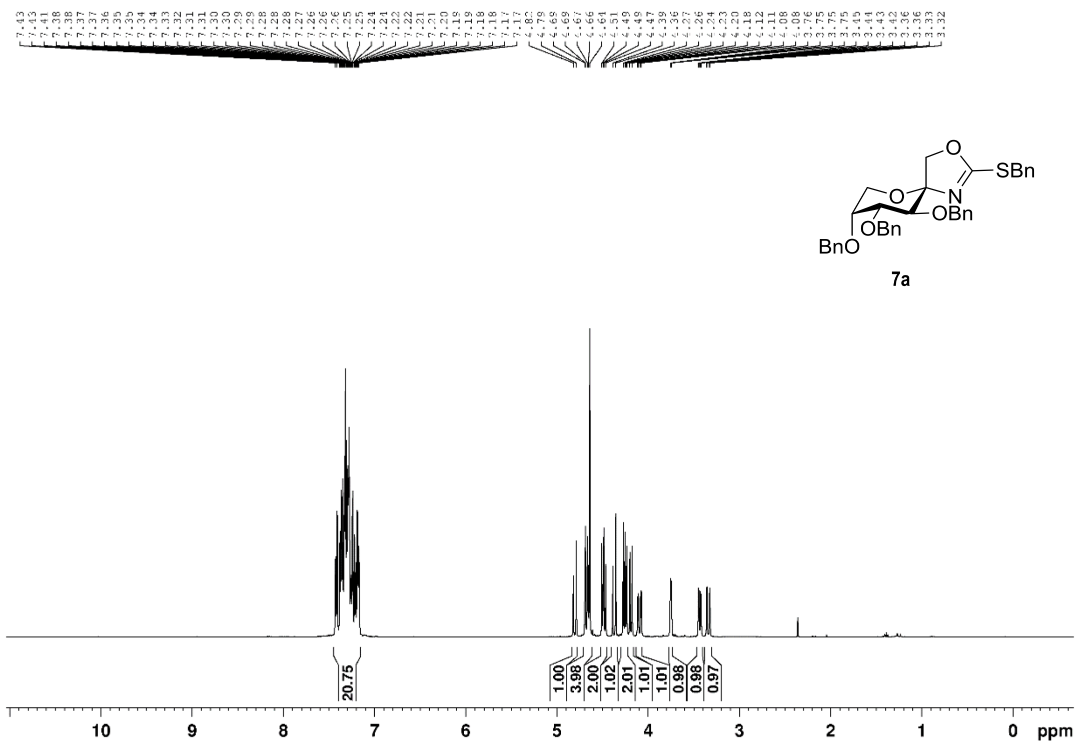
7.43 7.43 7.41 7.38 7.38 7.37 7.37 7.36 7.35 7.35 7.34 7.34 7.33 7.32 7.31 7.31 7.30 7.30 7.29 7.29 7.28 7.28 7.28 7.27 7.26 7.26 7.25 7.25 7.24 7.24 7.22 7.22 7.21 7.21 7.20 7.19 7.19 7.18 7.18 7.17 7.17
4.82 4.79 4.69 4.67 4.66 4.61 4.51 4.49 4.49 4.47 4.39 4.36 4.27 4.26 4.24 4.23 4.20 4.18 4.12 4.11 4.08 4.08 3.76 3.75 3.75 3.75 3.45 3.44 3.43 3.42 3.36 3.36 3.33 3.32
O
SBn
N
OBn
OBn
BnO
7a
20.75
1.00 3.98 2.00 1.02 2.01 1.01 1.01 0.98 0.98 0.97
10 9 8 7 6 5 4 3 2 1 0 ppm

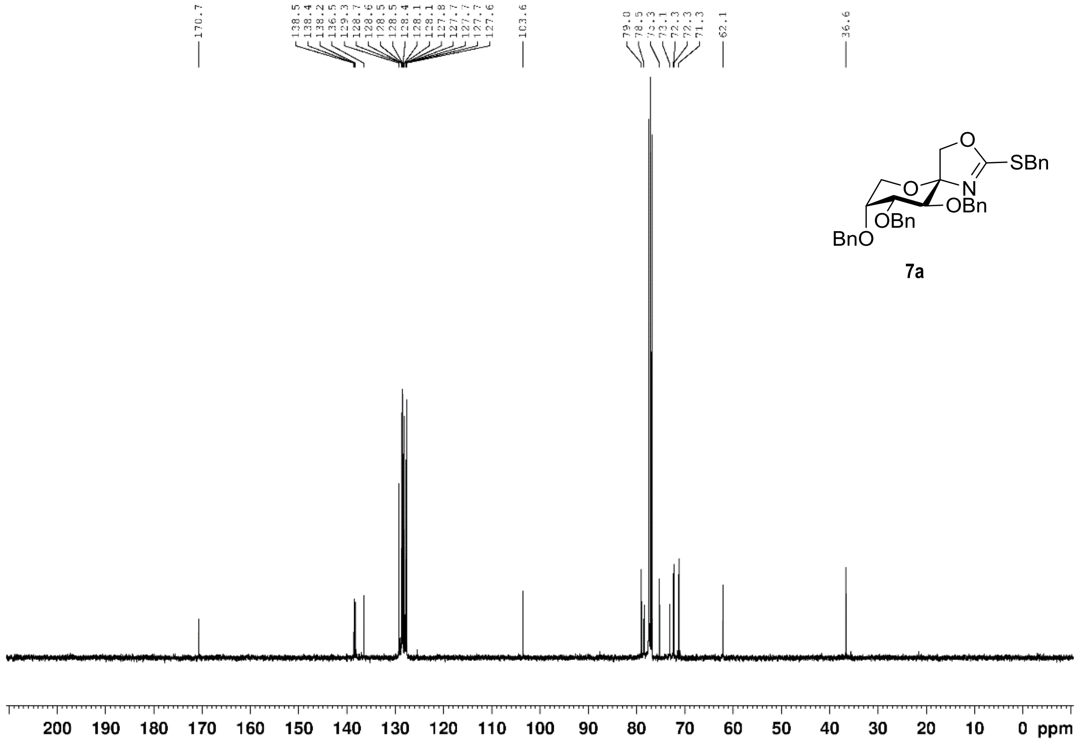
170.7
138.5 138.4 138.2 136.5 129.3 128.7 128.6 128.5 128.5 128.4 128.4 128.1 128.1 127.8 127.7 127.7 127.7 127.6
103.6
79.0 78.5 75.3 73.1 72.3 72.3 71.3
62.1
36.6
O
SBn
N
OBn
OBn
BnO
7a
200 190 180 170 160 150 140 130 120 110 100 90 80 70 60 50 40 30 20 10 0 ppm

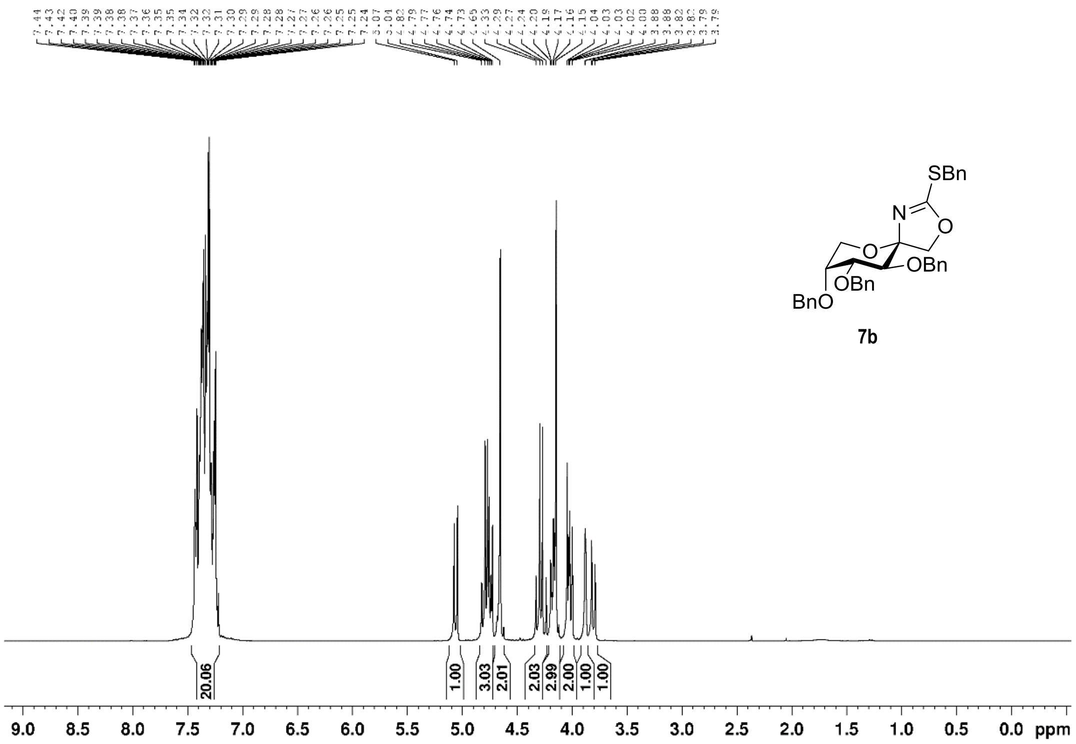
SBn
N
O
O
OBn
OBn
BnO
7b
20.06
1.00
3.03
2.01
2.03
2.99
2.00
1.00
1.00
9.0 8.5 8.0 7.5 7.0 6.5 6.0 5.5 5.0 4.5 4.0 3.5 3.0 2.5 2.0 1.5 1.0 0.5 0.0 ppm

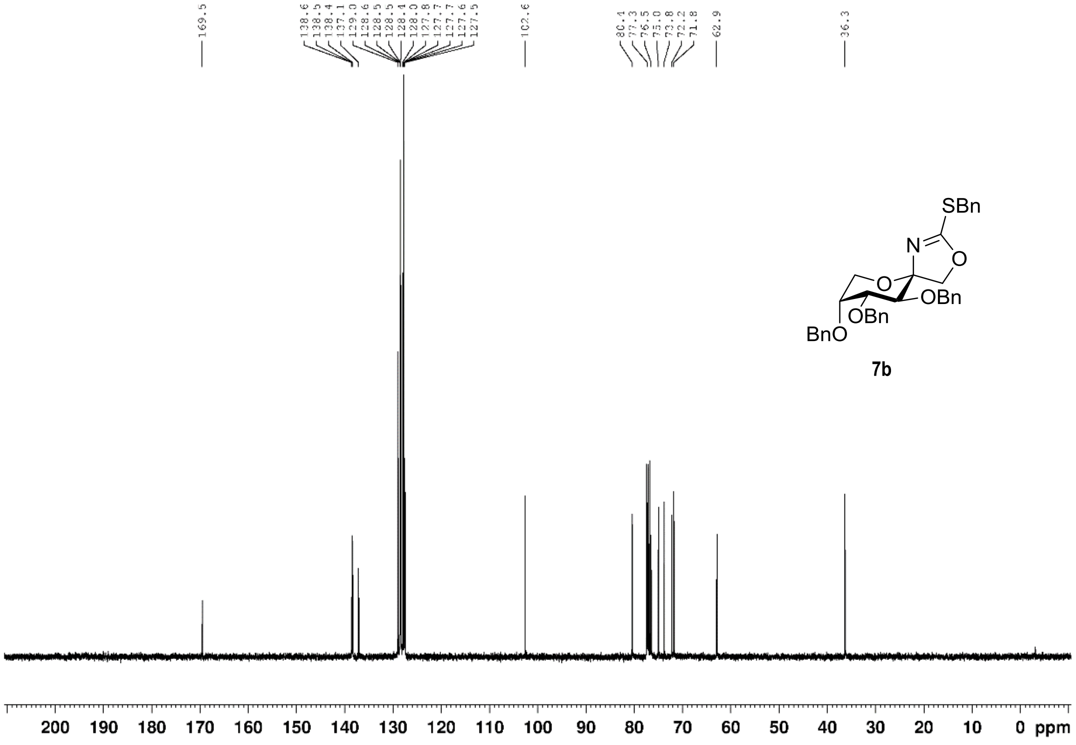
169.5
102.6
62.9
36.3
SBn
N
O
O
OBn
OBn
BnO
7b
200 190 180 170 160 150 140 130 120 110 100 90 80 70 60 50 40 30 20 10 0 ppm

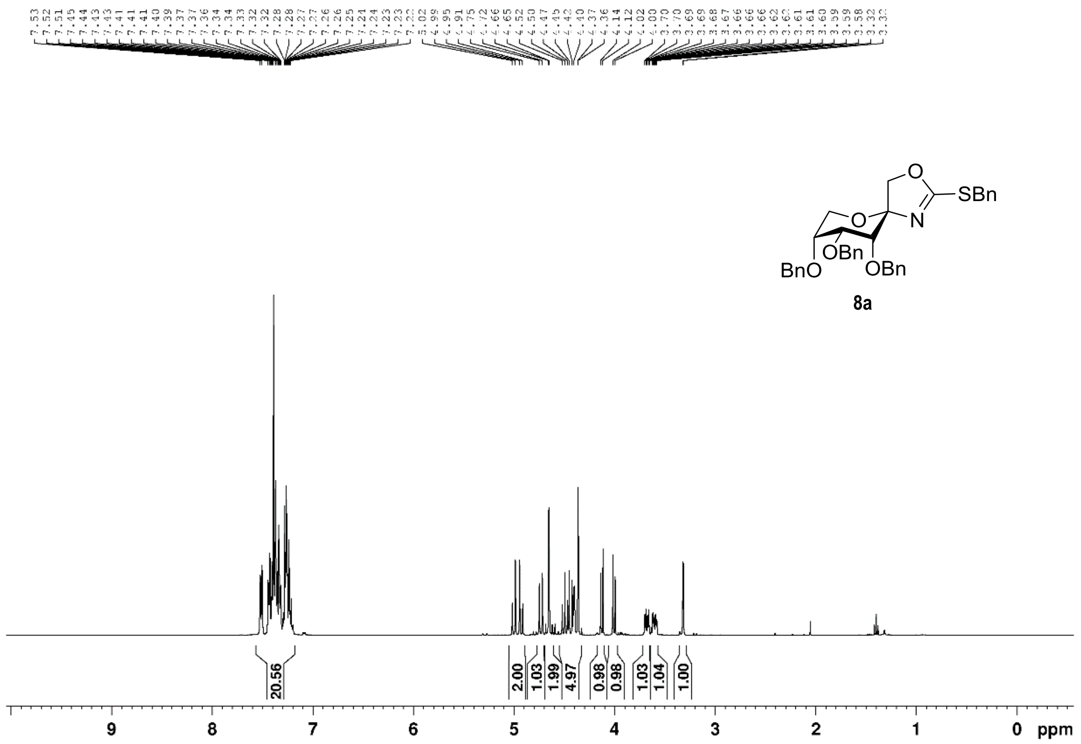
SBn
OBn
BnO
OBn
8a
ppm

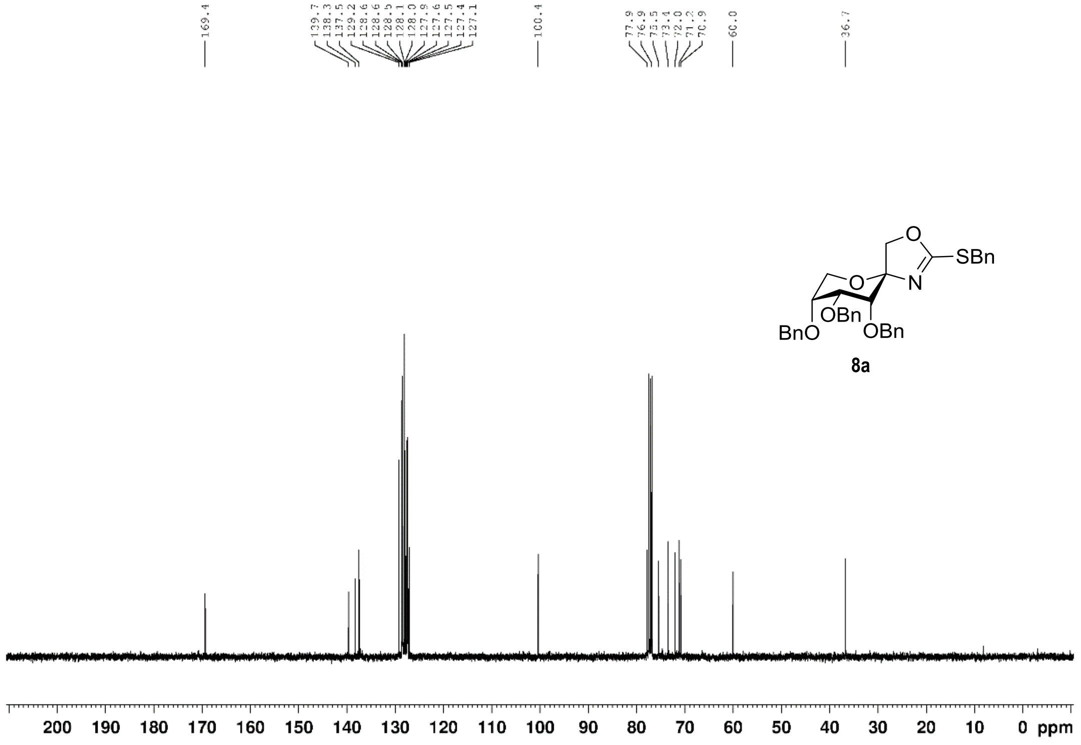
SBn
OBn
BnO
OBn
8a
ppm

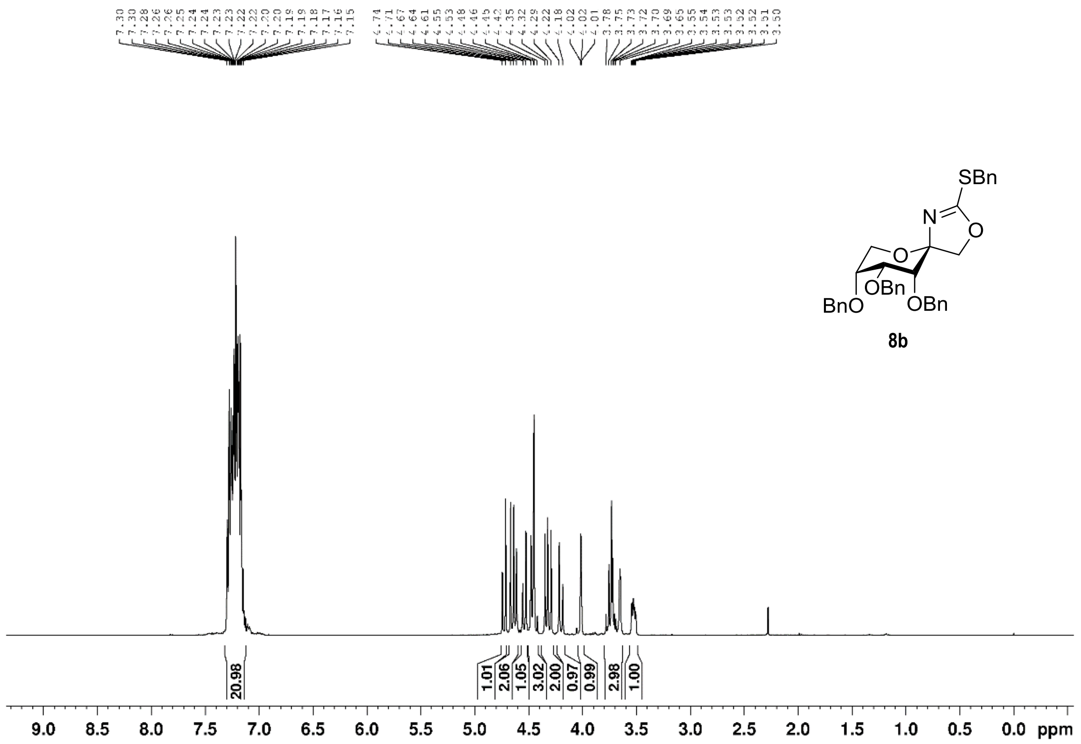
SBn
N
O
OBn
BnO
OBn
8b
20.98
1.01
2.06
1.05
3.02
2.00
0.97
0.99
2.98
1.00
9.0 8.5 8.0 7.5 7.0 6.5 6.0 5.5 5.0 4.5 4.0 3.5 3.0 2.5 2.0 1.5 1.0 0.5 0.0 ppm

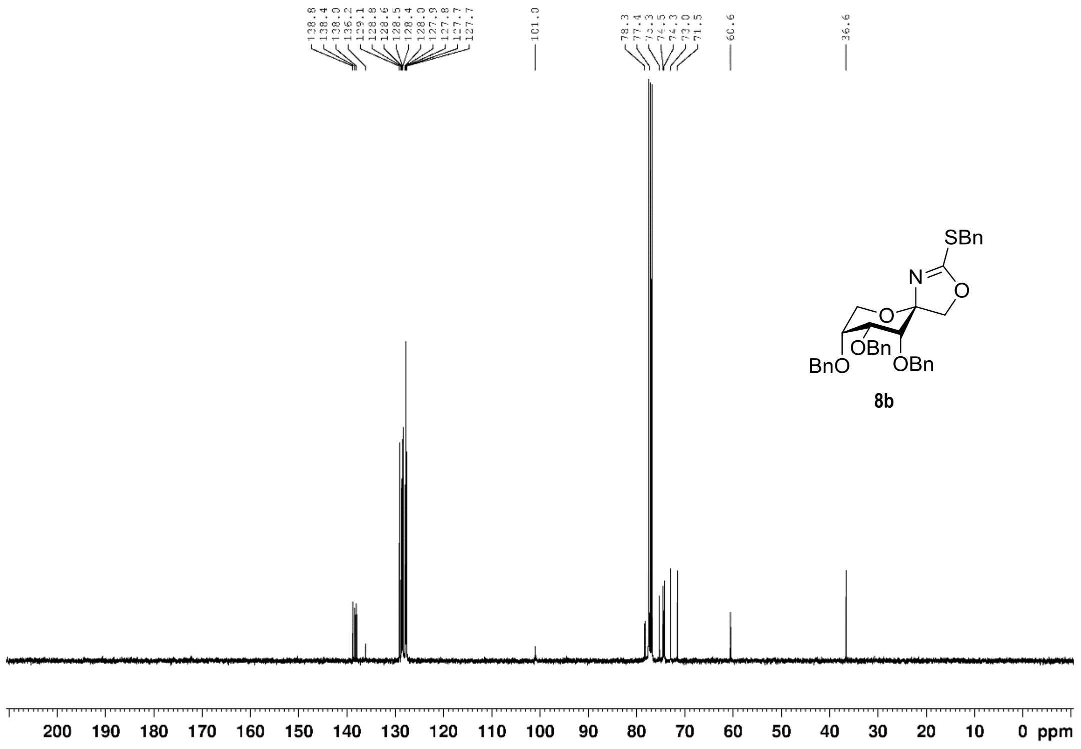
SBn
N
O
OBn
BnO
OBn
8b
200 190 180 170 160 150 140 130 120 110 100 90 80 70 60 50 40 30 20 10 0 ppm

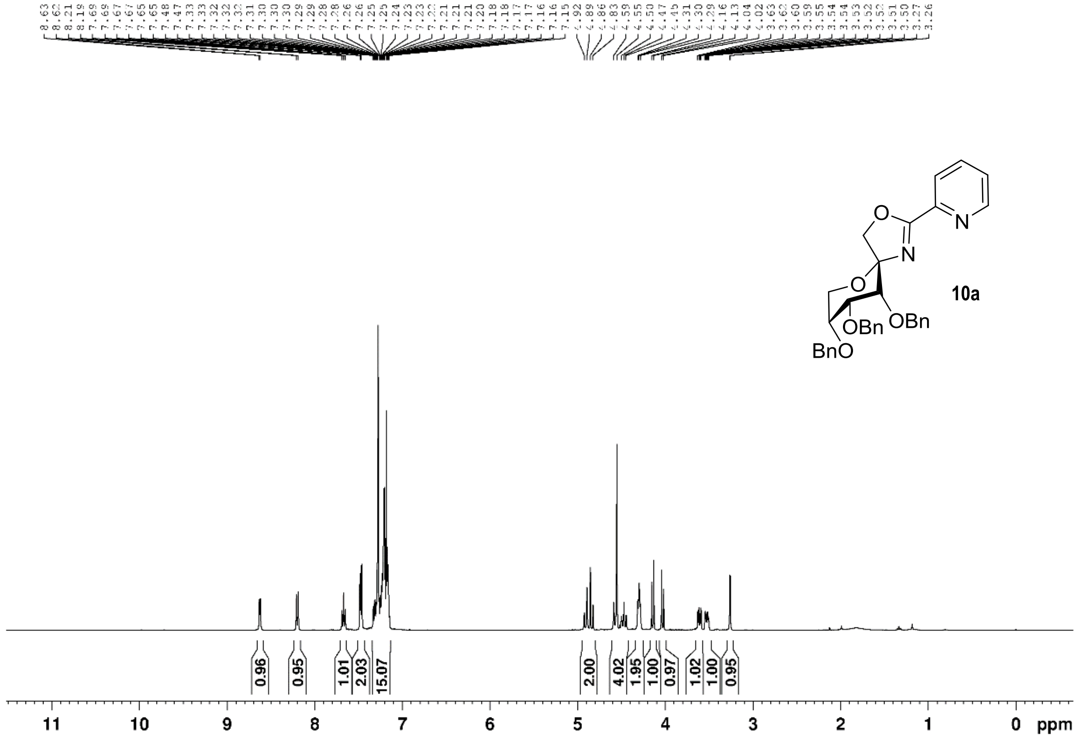
10a
OBn
OBn
BnO
ppm

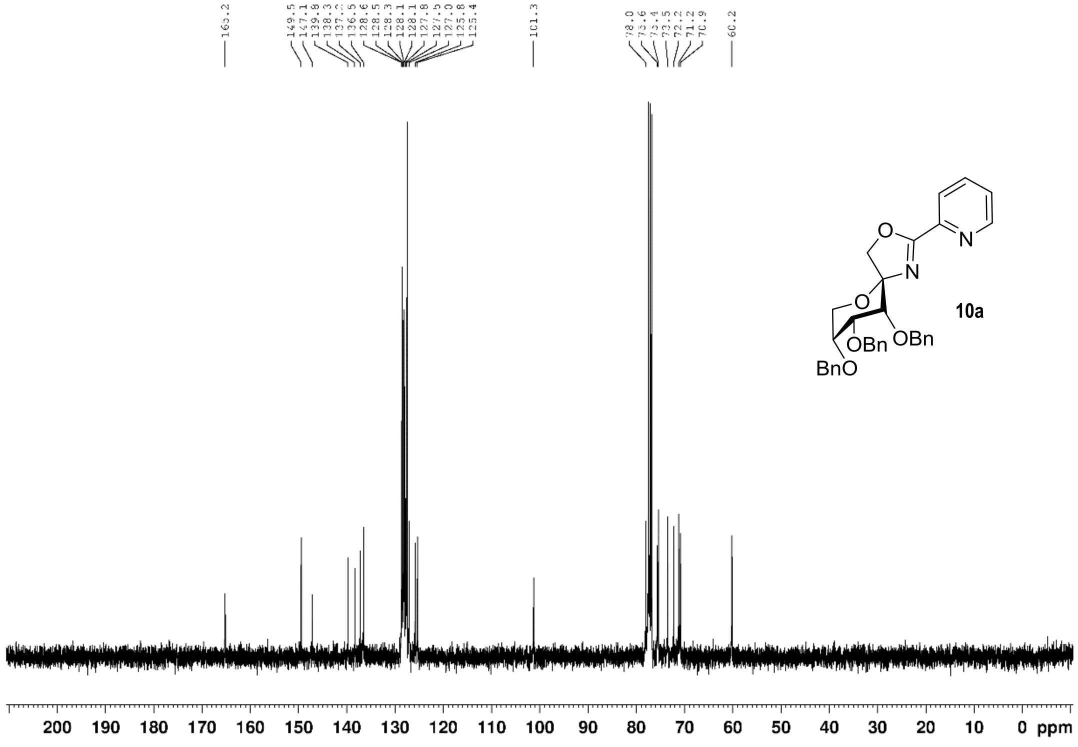
10a
OBn
OBn
BnO
ppm

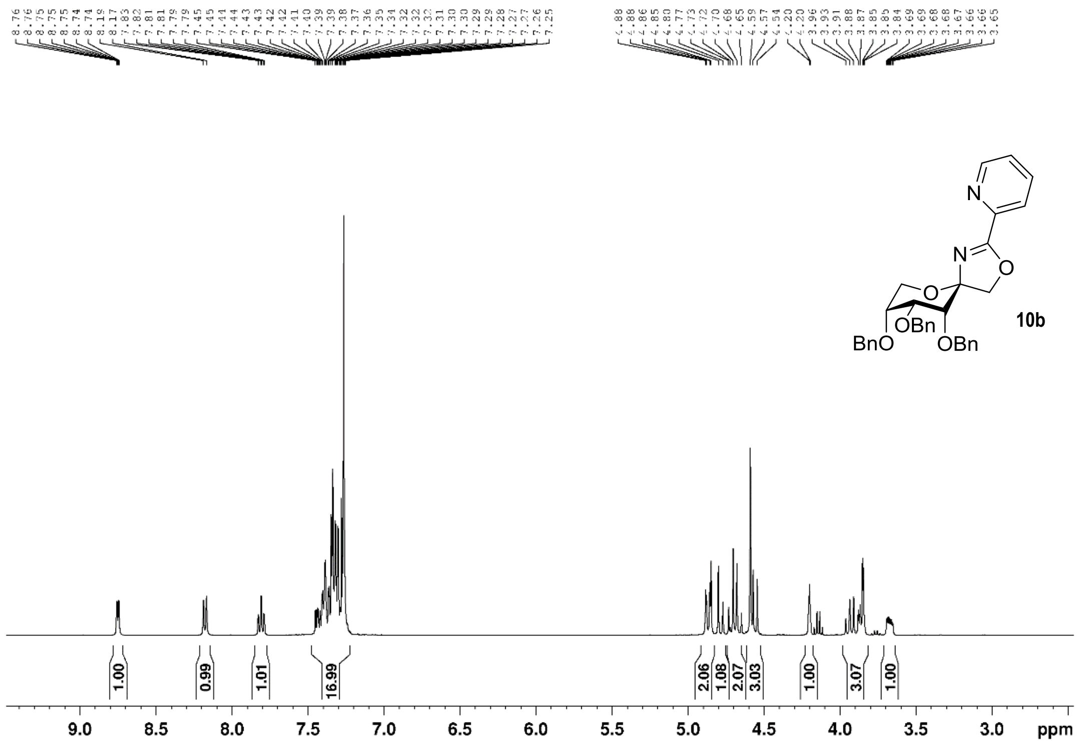
10b
BnO
OBn
OBn
1.00
0.99
1.01
16.99
2.06
1.08
2.07
3.03
1.00
3.07
1.00
9.0 8.5 8.0 7.5 7.0 6.5 6.0 5.5 5.0 4.5 4.0 3.5 3.0 ppm

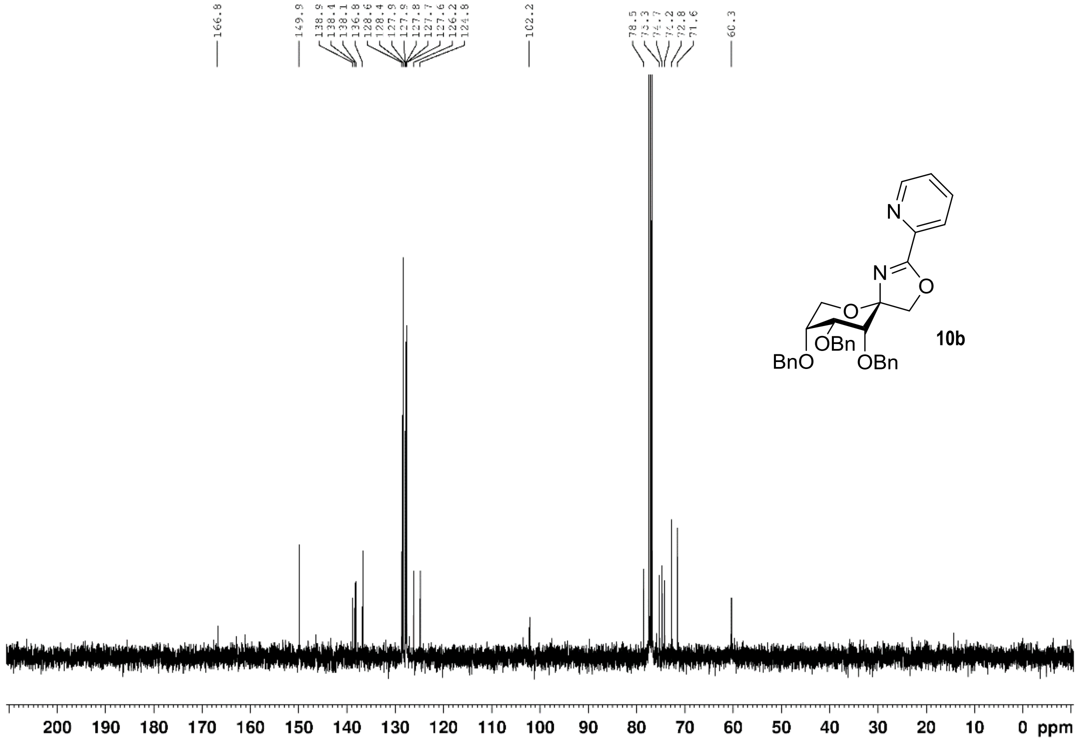
166.8
149.9
102.2
60.3
10b
BnO
OBn
OBn
200 190 180 170 160 150 140 130 120 110 100 90 80 70 60 50 40 30 20 10 0 ppm
